# A Comparison of Mobile and Fixed Device Access on User Engagement Associated With Women, Infants, and Children (WIC) Online Nutrition Education

**DOI:** 10.2196/resprot.6608

**Published:** 2016-11-15

**Authors:** John J Brusk, Robert J Bensley

**Affiliations:** ^1^ Western Michigan University Department of Human Performance and Health Education Kalamazoo, MI United States

**Keywords:** Internet, mobile phone, mHealth, eHealth

## Abstract

**Background:**

Online health education has expanded its reach due to cost-effective implementation and demonstrated effectiveness. However, a limitation exists with the evaluation of online health education implementations and how the impact of the system is attenuated by the extent to which a user engages with it. Moreover, the current online health education research does not consider how this engagement has been affected by the transition from fixed to mobile user access over the last decade.

**Objective:**

This paper focuses on comparing the impact mobile versus fixed devices have on user engagement key performance indicators (KPI) associated with the wichealth website (.org), an Internet-based parent-child feeding intervention offered to clients associated with the US Department of Agriculture’s Special Supplemental Nutrition Program for Women, Infants, and Children (WIC).

**Methods:**

Data were collected from 612,201 nutrition education lessons completed by 305,735 unique WIC participants in 21 states over a 1-year period. Data consisted of system-collected measures, profile items, and items from an exit survey administered at the conclusion of each lesson. User engagement was defined based on 3 KPIs associated with usage of the wichealth website: number of link views, link view time, and progression in stage of readiness to change. Independent samples *t* tests were used to compare KPIs between fixed only and mobile only device users and paired samples *t* tests were used to compare KPIs within users who completed at least one lesson each on both a fixed and mobile device. A logistic regression was performed to estimate the odds of KPI performance thresholds in the independent samples study group given access device type while controlling for confounding of user characteristics associated with these KPIs.

**Results:**

Analysis of 8 user characteristics (lessons completed, race, ethnicity, language, state of residence, pregnancy status, beginning stage of change, and preferred nutrition education method) were significantly (*P*<.001) related to various KPI differences between mobile and fixed device access. Non-mobile users were significantly (*P*<.001) more likely to engage based on all 3 KPIs, even after logistic regression control for the potential confounding related to the strongly associated user characteristics identified.

**Conclusions:**

The findings of this study support the idea that online health education developers need to seriously consider access device when creating programs. Online health education developers need to take extra effort to truly understand access patterns of populations being served, and whether or not access device will influence user engagement performance indicators.

## Introduction

Online health education, often referred to as electronic health (eHealth) and now mobile health (mHealth) education, has experienced tremendous growth over the last several years, primarily due to its cost-effectiveness [1]. The rapid growth of mobile broadband technology has expanded access to online health education among individuals with lower socioeconomic status whom may have had less access to fixed devices, such as a personal computer or laptop [2,3]. Not only has mobile broadband technology significantly extended the reach of the Internet, it has become the primary access method worldwide [4].

A recent systematic literature review of mobile nutrition apps concluded that effectiveness of mobile phone and tablet apps for online health education need additional research, as mobile platforms now allow consumers to access information on the go [5,6]. Mobile access to online health education represents a major shift in how users interact with information, resulting in differences in usage patterns and levels of engagement [7]. Many online health sites, including the wichealth website (.org), an online nutrition education and parent-child feeding behavior change system currently being implemented for use by the US Department of Agriculture’s (USDA) Special Supplemental Nutrition Program for Women, Infants, and Children (WIC) programs in 28 states and the platform of focus in this paper, began development when most access was fixed via device locations, such as personal computers at home, work, clinics, or libraries. Wichealth applies the eHealth Behavior Management Model in triaging a client through a series of response-dependent dialogue between the user and a virtual educator toward an initial stage of readiness to change a specific parent-child feeding behavior [8]. It is at this point where intervention content and resources are presented to the user. The client is then presented with the opportunity to engage in further stage-based resources based on intent to move toward active change. Further discussions of the model and features inherent within the wichealth system have been thoroughly described elsewhere [8,9]. However, mobile technology has made impressive gains in just the last several years. Ownership of cellular phones by US adults increased to 92% and mobile phones with app capabilities from 35% to 68% between 2011 and 2015 [10]. Even though mobile phone owners are more likely to be young, affluent, and highly educated, access is not restricted, as 52% of US adults earning less than US $30,000 per year own a mobile phone and mobile technology interest is high among WIC clients [10,11].

It is undeniable that the Internet has become a widely used resource for people seeking health information [12]. Online health education strategies can provide users with more flexibility and an opportunity to become more involved in the management of their health [13]. This increase in consumer knowledge may then lead to improved health outcomes, as online resources offer users greater interactivity and potential for engagement, which should enhance their learning and understanding [14-18]. Mobile access to health education, in particular, may at first appear beneficial for improving user engagement because people tend to be more proximally associated with their mobile devices, frequently keeping them close at hand. However, the quality of the engagement may actually suffer even though the impedance to engage is reduced and the frequency of engagement is increased. The manner in which mobile device users interact with Internet content is sufficiently different from those that access the media from a fixed device [19]. As a result, some evaluation researchers have called for alternative models to assess user impact as, despite the increase in usefulness that accompanies mobile access, this benefit has come at the expense of their usability in some contexts [20].

Over a decade ago, Zhang and Adipat [21] highlighted a number of usability challenges that occurred as a result of the advent of mobile devices, including the ability of users to access the Internet in multiple locations while doing multiple activities, smaller screen size, variable screen resolutions, more restricted user input, and limited processing power. More recent studies have demonstrated these challenges, especially with regard to user interaction. Harrison and colleagues [20] indicated that most usability research does not consider the impact of the mobile transition and its consequences. Their research addressed how mobile devices increase an individual’s cognitive load capacity. The increased ability for users to “multi-task” through mobile device use may come at a cost of user engagement with the content of interest, which is often a critical factor to the success or failure of an application [22]. In addition, mobile access changes the quality of user interaction with health and personal information. A study of data quality in Web surveys found mobile access generally produced lower item completion rates and shorter length of answers [23]. Similarly, a study comparing the differences in survey response completion quality and time found data collection via a mobile device was associated with longer user input time and lower quality and quantity responses. This may suggest that mobile device users either find it more difficult to input data into the online health education system or they may be accessing the system while performing multiple tasks, giving less than their full attention to the task at hand [24]. Furthermore, the effect of primacy is stronger in a mobile setting. Users are significantly more likely to select the top sorted link on a mobile device compared to a computer [25]. Even though it has been clear that Internet interaction differs depending on access device, a systematic review of 8 mobile technologies including mobile phones, personal digital assistants (PDA), PDA phones, enterprise digital assistants (EDA), portable media players, handheld video gaming consoles, and tablets screened from over 26,000 possible studies for inclusion concluded that the overall impact and effectiveness of the applications did not address mobile access as a potential barrier and critical design consideration for online health interventions [26].

More research is needed to determine the extent to which mobile access to Web applications may engage the user differently than fixed access devices and how to design applications to ensure this impact does not affect quality of the intervention. Few studies have been conducted that differentiate fixed eHealth and mHealth education, which is slowly becoming ubiquitous health (uHealth), as devices such as watches, eyeglasses, and home appliances will all soon be tapped into the Internet [2,3,5,13,27,28]. The purpose of this study was to expand the body of research exploring the differences between eHealth and mHealth engagement by exploring the usage pattern differences and impact on key performance indicators (KPI) between fixed and mobile device completion of parent-child feeding lessons associated with the wichealth website.

## Methods

### Participants

The population of interest for this study consisted of clients of the WIC program from 21 states who completed a lesson on the wichealth website during the government fiscal year period October 1, 2014 through September 30, 2015. Participants self-selected to complete a wichealth lesson as a means of meeting secondary contact requirements associated with the WIC program. Data collection protocols using wichealth have been approved for use by the Western Michigan University Human Subjects Institutional Review Board. Online informed consent was available prior to completion of the online survey.

### Data Collection

Data utilized in this study was garnered from 305,735 unique WIC clients who completed 612,201 wichealth lessons over the 1-year period of study. Participants were divided into 2 study groups. The first study group consisted of 280,845 unique WIC clients whose interaction with the wichealth website during the study period consisted of either fixed (desktop computer, laptop, or kiosk) or mobile (phone or tablet) device access, but not both. The second study group consisted of 24,890 unique WIC clients who completed both at least one lesson using fixed access and at least one lesson using a mobile device during the study period. All lessons were completed using the wichealth website, which consists of a responsive design that adjusts based on screen size of device. Data consisted of 6 system-collected measures (links viewed, link view time, device type, beginning and ending stages of change, lessons completed), 5 profile items (ethnicity, race, language, pregnancy status, state of residence), and 1 item focused on nutrition education method (“How do you prefer to get your nutrition education”) from an exit survey administered at the conclusion of each lesson. User engagement was defined based on 3 KPIs associated with wichealth usage, including number of link views, link view time, and progression in stage of change. Link visits are a central component of the behavior change theory inherent within the wichealth system, as it is at the link level where stage-based content and skills are delivered. Links consist of static and interactive webpages, downloadable Portable Document Folders (PDFs), and videos where content and skills relevant to the behavioral focus of the lesson are presented. All links are selected and developed based on learning and behavior change skills relevant to the priority population. Reliability of the exit survey was previously established using Cronbach alpha, and the staging algorithms used to identify beginning and ending stages were based on criteria previously used to determine stages of change and have been described in detail elsewhere [8,9,29]. Separate studies to determine current stage of change associated with parent-child feeding behaviors among a sample of WIC participants from Michigan and Washington found similar trends as the staging algorithms used in the wichealth website, further increasing confidence in the validity of data collection procedures used in the current study [30].

### Statistical Analysis

The purpose of this study was to determine how wichealth KPIs varied between fixed and mobile device access. First, user characteristics were evaluated to identify whether they were independently associated with either the KPI outcomes or device type. Independent samples *t* tests were used to compare KPIs between fixed only and mobile only device users and paired samples *t* tests were used to compare KPIs within users who completed at least one lesson each on both a fixed and mobile device. A logistic regression was performed to estimate the odds of KPI performance thresholds in the independent samples study group given access device type while controlling for confounding of user characteristics associated with these KPIs. Odds ratios (OR) with significance determined using chi-square were calculated for both study groups using the general linear models package in R. Per the American Statistical Association [31], using a *P* value by convention, such as achieving statistical significance when *P*<.05, does not ensure a material effect and is likely to generate a number of false and weak claims about a relationship. Given this and the very large sample size available for this research, only levels of significance below .001 were reported for *P* values obtained from results of Student *t* tests and chi-square. This was to ensure differences that are not practically important or relevant were not considered as such.

## Results

Mobile access made up 43.66% (267,317/612,201) of all wichealth lessons completed during the study period. Access to the wichealth website by a mobile device was inversely associated with user engagement, in particular the number of educational links viewed within a wichealth lesson and progression of stage of readiness to change. Individuals who accessed wichealth using a mobile device were more than 2 times less likely to visit any educational links that are part of the wichealth lesson. Those who did access a link via a mobile device accessed, on average, fewer links and spent fewer minutes viewing those links than non-mobile device users. With regard to intent to change the parent-child feeding behavior associated with the lesson, mobile device users who began a lesson in an early stage of readiness to change (precontemplation, contemplation, or preparation) were significantly less likely to progress in stage of change than users who accessed wichealth via a personal computer or kiosk (Table 1).

Although these differences in wichealth KPIs appear to be statistically significant (*P*<.001) between fixed and mobile usage, several user characteristics were also found to be associated with the wichealth KPIs. The paired sample study group of individuals who completed lessons via both fixed and mobile access should control for this confounding; however, this group of users is not defensibly generalizable to the group of individuals that only completed lessons via either fixed or mobile access, but not both. Number of lessons was not used as a measure of KPI because the typical user only completed a single lesson. Within the group that completed lessons only on a fixed or mobile device but not both, 56.56% (158,835/280,845) completed only 1 lesson. Both fixed and mobile access users in this group averaged close to 1.9 lessons per user, which was not significantly different. Users who completed lessons via both fixed and mobile access represented individuals that were likely more engaged, as they completed at least 2 lessons, whereas the typical user completed less than 2 lessons. Further, observed differences between some KPIs of users that completed at least 2 lessons via either fixed or mobile access and users that completed at least 2 lessons via a combination of fixed and mobile access were significantly different (*P*<.001) indicating that these groups are not representative of each other (Table 2).

**Table 1:** Key performance indicators by device type.

Performance indicator	Independent samples		Paired samples	
	Fixed	Mobile	Fixed	Mobile
Unique users, n	161,356	119,489	12,445	12,445
Lessons completed, n	303,815	227,273	41,069	40,044
LLV^a^, %	75.23	32.56^c^	74.95	40.66^c^
Link views per LLV, n	2.18	1.76^c^	2.25	2.01^c^
Link view minutes per LLV, n	1.46	0.84^c^	1.61	1.32^c^
ESOC^b^, n	98,777	67,221^c^	13,327	12,468
ESOC with stage progression, %	85.55	80.89^c^	84.95	84.41

^a^LLV: lessons completed that had at least one link view.

^b^Lessons beginning in an early stage of change (ESOC).

^c^*P*<.001.

**Table 2:** Key performance indicators by lessons completed.

Performance indicator	Independent samples		Paired samples: 2 or more lessons completed
	1 lesson completed	2 or more lessons completed	
Unique users, n	158,835	122,010	24,890
Lessons completed, n	158,835	372,253	81,113
LLV^a,^ %	55.71^c^	57.51	58.02
Link views per LLV, n	1.89^c^	2.15	2.17
Link view minutes per LLV, n	2.46	0.83^c^	1.51
ESOC^b^, n	54,098	111,900	25,795
ESOC with stage progression, %	85.20	82.86^c^	84.69

^a^LLV: lessons completed that had at least one link view.

^b^Lessons beginning in an early stage of change (ESOC).

^c^*P*<.001.

Other user characteristics associated with wichealth KPIs included race, Hispanic ethnicity, language, state, pregnancy status, early beginning readiness to change status, and preferred method for receiving nutrition education. Lessons with link views, links viewed per lesson, and link view time demonstrated some significant differences by race (Table 3). Although black users had a similar proportion of completed lessons with link views as white users, those who did have link views, had fewer on average than other users. They were also less likely to advance in stage of change. Users who did not report being either white or black were less likely to view a link during their lesson, but more likely to spend more time on the links that were accessed. Similarly, users of Hispanic ethnicity were less likely to access a link during their lesson compared to other users, but those who did access links also viewed them for longer. Hispanic users were also more likely to advance in stage of change (Table 4). These findings raise the suspicion that users who either did not report race or reported themselves as “other” may have actually considered their race to be Hispanic, as has been found elsewhere [32]. Hispanic engagement in KPI is further demonstrated as users of the Spanish language version of wichealth consistently had more link views and link view time among individuals who used at least one link during their lesson compared to English version users (Table 5).

**Table 3:** Key performance indicators by race.

Performance indicator	Independent samples			Paired samples			
	White	Black	Other/missing	White	Black	Other/missing
Unique users, n	145,853	36,201	98,791	12,676	2730	9484
Lessons completed, n	274,624	65,535	190,929	40,274	9154	31,685
LLV^a^, %	58.99	59.38	53.23^c^	58.80	62.67^c^	55.72
Link views per LLV, n	2.08	1.86^c^	2.14	2.17	1.92	2.25
Link view minutes per LLV	1.28	1.23	1.38^c^	1.47^c^	1.33	1.62^c^
ESOC^b^	86,123	20,467	59,408	12,916	2907	9972
ESOC with stage progression, %	84.49	78.28^c^	84.29	85.44	79.86^c^	85.09

^a^LLV: lessons completed that had at least one link view.

^b^Lessons beginning in an early stage of change (ESOC).

^c^*P*<.001.

**Table 4:** Key performance indicators by ethnicity.

Performance indicator	Independent samples		Paired samples	
	Non-Hispanic	Hispanic	Non-Hispanic	Hispanic
Unique users, n	184,023	96,822	15,190	9700
Lessons completed, n	348,018	183,070	48,871	32,242
LLV^a^, %	59.45	52.25^c^	59.77	55.36^c^
Link views per LLV, n	2.06	2.10	2.12	2.25^c^
Link view minutes per LLV	1.25	1.44^c^	1.42	1.67^c^
ESOC^b^	109,474	56,524	15,544	10,251
ESOC with stage progression, %	82.98	85.04^c^	84.16	85.53^c^

^a^LLV: lessons completed that had at least one link view.

^b^Lessons beginning in an early stage of change (ESOC).

^c^*P*<.001.

**Table 5:** Key performance indicators by language.

Performance indicator	Independent samples		Paired samples	
	English	Spanish	English	Spanish
Unique users, n	268,655	12,189	24,074	816
Lessons completed, n	508,050	23,038	78,053	3060
LLV^a^, %	57.13	53.52^c^	57.84^c^	62.58
Link views per LLV, n	2.06	2.46^c^	2.12	3.40^c^
Link view minutes per LLV	1.29	1.83	1.49	1.87^c^
ESOC^b^	158,140	7858	24,704	1091
ESOC with stage progression, %	83.47^c^	86.42	84.86	80.73^c^

^a^LLV: lessons completed that had at least one link view.

^b^Lessons beginning in an early stage of change (ESOC, precontemplation, contemplation, preparation).

^c^*P*<.001.

**Table 6:** Key performance indicators by state mobile access level.

Performance indicator	Independent samples		Paired samples	
	High	Low	High	Low
Unique users, n	206,274	74,571	20,156	4734
Lessons completed, n	392,614	138,474	66,161	14,952
LLV^a^, %	55.15	62.13^c^	57.38	60.83^c^
Link views per LLV, n	2.05	2.14	2.18	2.11
Link view minutes per LLV	1.31	1.32	1.51	1.50
ESOC^b^	121,628	44,370	20,882	4913
ESOC with stage progression, %	82.96	82.65	84.22	84.14

^a^LLV: lessons completed that had at least one link view.

^b^Lessons beginning in an early stage of change (ESOC).

^c^*P*<.001.

**Table 7:** Key performance indicators by pregnancy status.

Performance indicator	Independent samples		Paired samples	
	Not pregnant	Pregnant	Not pregnant	Pregnant
Unique users, n	237,117	43,728	19,891	4999
Lessons completed, n	439,654	91,434	63,046	18,067
LLV^a^, %	57.47	54.54^c^	58.56	56.11^c^
Link views per LLV, n	2.06	2.15	2.09	2.46^c^
Link view minutes per LLV	1.33	1.19^c^	1.54	1.41^c^
ESOC^b^	139,199	26,799	20,223	5572
ESOC with stage progression, %	85.06	76.28^c^	87.42	74.82^c^

^a^LLV: lessons completed that had at least one link view.

^b^Lessons beginning in an early stage of change (ESOC).

^c^*P*<.001.

User state of residence was grouped based on whether mobile access rates in that state were high or low given the relative extent of usage in the state compared to other participating states. Alabama, California, Iowa, Louisiana, Michigan, and South Dakota all had mobile access rates that significantly exceeded the overall average of 43.66%. These states were assigned a high level of mobile access, while the remaining were classified as low. States that tended to have lower mobile access levels were more likely to have users that used at least one link view during their lesson (Table 6).

Pregnancy status was strongly associated with wichealth KPIs, with pregnant users significantly less likely to complete lessons with at least one link view, spend time on links accessed, and progress in stage of change than non-pregnant users (Table 7). These findings may be related to the fact that users completed pregnancy-specific lessons at a greater rate than other lessons. This set of lessons address behaviors that are often more difficult for users to progress along the stage of change continuum, and therefore the cause of the lower level of progression is likely related to the lesson, rather than the user.

User beginning stage status is another characteristic associated with wichealth KPI performance. Specifically, early stage of readiness to change users were more likely to use a link during their lesson, and they accessed about one link more on average than non-early stage of readiness to change users (Table 8). This makes sense because when users progress, they are provided the opportunity to continue their learning with an additional pool of links from which to select in order to help them progress further.

Finally, user preference for wichealth as a means for receiving future nutrition education was assessed for its association with wichealth KPIs in each study group. Users who preferred the wichealth website were more likely to view more links during their lesson and to progress in stage of readiness to change than users who preferred another nutrition education method, such as counseling, group classes, or other onsite learning activities (Table 9).

**Table 8:** Key performance indicators by early begin stage user.

Performance indicator	Independent samples		Paired samples	
	Non-ESOC^a^	ESOC	Non-ESOC	ESOC
Unique users, n	153,862	126,983	14,408	10,482
Lessons completed, n	233,647	297,441	39,721	41,392
LLV^b^, %	53.19	59.94^c^	54.21	61.67^c^
Link views per LLV, n	1.49	2.48^c^	1.59	2.65^c^
Link view minutes per LLV	1.36	1.27	1.54	1.48
ESOC	N/A	165,998	N/A	25,942
ESOC with stage progression, %	N/A	83.66	N/A	84.69

^a^Lessons beginning in an early stage of change (ESOC).

^b^LLV: lessons completed that had at least one link view.

^c^*P*<.001.

**Table 9:** Key performance indicators by preferred nutrition education method.

Performance indicator	Independent samples		Paired samples	
	Other	wichealth	Other	wichealth
Unique users, n	54,478	226,367	4015	20,875
Lessons completed, n	85,380	445,708	11,794	69,319
LLV^a^, %	56.36	57.09	57.38	58.13
Link views per LLV, n	1.86	2.11^c^	2.00	2.20^c^
Link view minutes per LLV	1.41^c^	1.29	1.53	1.51
ESOC^b^	25,664	140,334	3617	22,178
ESOC with stage progression, %	77.43	84.83^c^	80.45	85.38^c^

^a^LLV: lessons completed that had at least one link view.

^b^Lessons beginning in an early stage of change (ESOC).

^c^*P*<.001.

Given the paired sample study group of individuals having completed a lesson via both fixed and mobile access, control of confounding user characteristics on the association of lower KPIs with mobile access was essentially achieved. Within this group, there was still significant differences between KPIs for lessons completed via fixed compared to mobile access, such as the percent of lessons completed using a link and the link view minutes per lesson; however, the main outcome of stage of change progression was not significant (see Table 1). This suggests that the effect of mobile device on user engagement was still significant in that there was a mobile-specific reason for lower engagement; however, mobile use did not appear to impact progression in stage of readiness to change. Yet as indicated, because this paired sample study group was not representative of the typical user, control for the user characteristics associated with wichealth KPIs is warranted to evaluate the effect on the observed difference in stage progression between typical fixed access and mobile access users. To achieve this, a logistic regression model was developed to include all of the user characteristics previously presented in order to determine if the associations observed of stage progression and lower wichealth KPIs among mobile users was a product of confounding or effect modification. The results of the logistic regression model set up with device access as the dependent outcome variable (fixed or mobile) and the wichealth KPIs as predictors along with all of the associated user characteristics is presented in Table 10. In this manner, the association of the wichealth KPIs and mobile access type could be evaluated controlling for any potential confounding or effect modification of user characteristics found to be related to the KPIs. Table 10 contains the regression coefficients and their standard errors, the *z* statistic, ORs, and confidence intervals (CIs). Supporting the univariate comparisons made above, all of the model predictors were statistically significant in their association with device type. The logistic regression coefficients can be interpreted as the change in the log odds of whether a mobile device was used for a 1-unit increase in the wichealth KPIs or user characteristic variable.

**Table 10:** Independent samples study group logistic regression model results.

Model feature	beta^a^	SE^b^	*z* ^c^	OR^d^	95% CI^e^ (upper-lower)
Intercept	.159	0.205	6.13^g^	1.17	1.11-1.23
Race (black)	.695	0.013	52.16^g^	2.00	1.95-2.06
Race (other)	.064	0.011	6.15^g^	1.07	1.04-1.09
Hispanic	.286	0.011	26.52^g^	1.33	1.30-1.36
Language	.184	0.022	8.39^g^	1.20	1.15-1.25
State mobile access	.306	0.010	30.18^g^	1.36	1.33-1.38
Pregnancy status	.030	0.012	2.39	1.03	1.01-1.05
Preferred nutrition education	.026	0.011	1.90	1.02	1.00-1.04
ESOC^f^	–.093	0.016	–5.31^g^	0.92	0.89-0.95
Link view	–1.719	0.010	–178.19^g^	0.18	0.17-0.18
Link view minutes	–.205	0.005	–36.54^g^	0.82	0.81-0.82
Stages progressed	–.201	0.038	–5.35^g^	0.82	0.76-0.88

^a^beta: regression coefficient.

^b^SE: standard error.

^c^*z*: z statistic.

^d^OR: odds ratio.

^e^CI: confidence interval.

^f^Lessons beginning in an early stage of change (ESOC).

^g^*P*<.001.

After controlling for user characteristics associated with mobile device use, users of mobile devices were over 5 times less likely to access any links during their lesson (OR = 0.18, *P*<.001, 95% CI [.17, .18]). Further, mobile device users were less likely to spend as many minutes viewing links when they did use them (OR = 0.82, *P*<.001, 95% CI [.81, .82]). Finally, even with all potential confounders accounted for in the model, the stages progressed among early beginning stage of change users was significantly lower among those accessing wichealth via mobile rather than fixed access (OR = 0.82, *P*<.001, 95% CI [.76, .88]).

## Discussion

### Principal Findings

The advent and expansion of mobile devices has clear implications for Internet intervention designers. As demonstrated in this study, the expansion of mHealth use in the wichealth website, which was originally designed for completion on a fixed device, resulted in lower KPIs. Based on the findings presented, it is clear that a difference exists between mobile and fixed device users in how they interact with this online nutrition education and behavior change system.

Although the review of literature previously presented indicates a number of reasons why mobile devices often achieve lower levels of performance associated with measures of engagement, strategies for how to address these issues, especially with respect to wichealth, are not clear. The observation that user engagement is impeded by mobile device use across many user characteristics such as age, race, language, state of residence, and preference for the learning modality, demonstrates how strong this impact is and underlines the significance of implementing design features to diminish it. In fact, all key wichealth performance measures were significantly lower for mobile device users.

The findings of this study support the idea that online health education developers need to seriously consider access device when creating programs. Over the next year it is likely wichealth will transition to become accessed primarily by mobile devices, as personal computers and kiosks become a less frequent option for individuals to retrieve online content. Mobile access of wichealth lessons has been increasing by 15% every 6 months, which has now made wichealth predominately accessed via mobile device. This transition has important implications, especially as users who access wichealth via a mobile device behave in a significantly different manner than users accessing a lesson by a computer, laptop, or kiosk. To address the findings presented in this study, the developers of wichealth recently redesigned the experience to ensure it is appropriate for the growing percentage of mobile users. A mobile first design strategy was used to ensure the responsive nature of the website did not deteriorate on mobile devices. Specific design changes with wichealth will be described elsewhere, as the purpose of this study was to present findings that would raise awareness in developers to ensure mobile user engagement characteristics are not automatically lumped together with fixed device users, but rather design focuses on both methods of access in order to create the most likely positive user experience. It is important for developers to consider the nature of the mobile access environment. Mobile phones and tablets are indicative of “on the go” usage, whereas access from a fixed device may be associated with users having more time and a better environment for focusing on the intervention. Further, mobile devices have less screen viewing real estate, which may increase the likelihood that users will not be as engaged. Finally, mobile devices may be less likely to be fully compatible with the internet content presented, lowering measures of user engagement.

### Limitations

Results should be interpreted realizing limitations existed. Wichealth was originally conceived as a fixed device intervention although it incorporated a responsive design appropriate for a mobile experience. As such, generalizability of results should be considered with this in mind. Another potential limitation is that participation in wichealth was through self-selection versus assignment, reducing the ability to generalize findings to all WIC populations. In addition, historically approximately 40% of wichealth lessons tended to have been completed by repeat users, which may have influenced findings. However, it is not conclusive whether repeat users always used the same access device for more than one lesson. Even so, the large number of users and lessons completed within this study mitigate any extreme influence a few users may have had on findings.

### Recommendations

There are many opportunities for further study, as this description of wichealth use has generated many questions and areas of speculation. First it is interesting that some key user characteristics such as Spanish language, black race, and user state of residence in Alabama were all associated with a higher likelihood that the user completed their lesson using a mobile device. Future research could attempt to address how this may be related to whether mobile device Internet access was the initial means for these users to gain access to the Internet on a regular basis. Also, these users appeared to be less impacted in terms of wichealth KPIs compared to fixed and mobile access users.

Additional investigation into whether the device operating system has any impact on measures of user engagement is warranted. For example, is there a difference in how these measures are affected if the user has an Android or iOS platform? Also, many of the reasons speculated for why mobile device access may have lower levels of user engagement could be evaluated by comparing mobile phone and tablet access, both of which were considered mHealth devices. As more users completed their lessons using a mobile device, additional investigation of these subcategories of mobile device usage should be completed.

### Conclusions

Online health education developers need to take extra effort to truly understand access patterns of populations being served, and whether or not access device will influence user engagement performance indicators. As mobile access continues to increase, especially among younger populations, application managers need to consider what changes in design and functionality needs to occur to ensure the intervention being delivered is appropriate for the user.

## Abbreviations

CI: confidence interval
EDA: enterprise digital assistant
ESOC: early beginning stage of readiness to change
eHealth: electronic health
KPI: key performance indicators
LLV: lessons completed that had at least one link view.
mHealth: mobile health
OR: odds ratio
PDA: personal digital assistant
uHealth: ubiquitous health
USDA: US Department of Agriculture
WIC: Special Supplemental Nutrition Program for Women, Infants, and Children
